# Pet ownership issues encountered by geriatric professionals: Preliminary findings from an interdisciplinary sample

**DOI:** 10.3389/fpsyg.2022.920559

**Published:** 2022-09-14

**Authors:** Jessica Bibbo, Justin Johnson, Jennifer C. Drost, Margaret Sanders, Sarah Nicolay

**Affiliations:** ^1^Center for Research and Education, Benjamin Rose Institute on Aging, Cleveland, OH, United States; ^2^Senior Health, Complex Care Institute, Summa Health, Akron, OH, United States; ^3^Family & Community Medicine, Northeast Ohio Medical University, Rootstown, OH, United States

**Keywords:** pets, older adult, dementia, caregiving, pet owner, geriatric patients, care partner, geriatric workforce

## Abstract

Pets often factor in older adults’ health behaviors and decisions. However, the degree to which issues related to pet ownership are encountered or addressed by professionals working with this population remains unknown. The aim of this study was to identify specific issues stemming from pet ownership professionals had encountered in their work with older adults, people living with dementia, and care partners. An interdisciplinary (e.g., social services and healthcare) sample (*N* = 462, 89.13% female, *M*_*age*_ = 53.02, *SD*_*age*_ = 12.18) completed an online survey addressing pet ownership issues encountered in their work. Descriptive statistics, t-tests, and repeated measures ANOVAs were used to analyze quantitative data. A conventional content analysis was used to analyze open-ended responses to an item asking about “other” issues encountered in their work with these populations. The professionals estimated 46.29% of clients had been pet owners, 41.23% regularly asked about pets, and 79.22% had encountered issues related to pet ownership. Specific issues raised to the professionals varied by type of client. The professionals reported older adults most often raised getting pet items into the home and concerns about their pets’ health. The issues most often raised by people living with dementia to the professionals were planning for the pet due to a housing transition and basic pet care. Care partners focused on basic pet care and planning for the pet due to a housing transition. The professionals themselves most often raised the issues of basic pet care, concerns about falling, and the pets’ behavior. Professionals who entered clients’ homes were more likely to raise issues stemming from pet ownership compared to those who reported they did not enter clients’ homes in their current job, *t*(429.40) = 5.59, *p* < 0.00001. The eleven new issues identified by the content analysis (e.g., pets impeding care, people refusing care due to the pet) underscored how the health and wellbeing of people and their pets are linked. The results of this study provide strong evidence that professionals do encounter issues related to pet ownership. Including issues stemming from pet ownership into procedures, policies, and programs is likely to have positive impacts on those served by and working in the geriatric workforce.

## Introduction

Healthy aging focuses on maintaining wellbeing through the creation of environments which allow individuals to enact their values and preferences throughout life (World Health Organization [WHO], 2021). Individuals’ functional abilities include their physical, cognitive, and psychological capacities, along with their environment, and the interaction between the individual and their environment. Healthy aging is not living without disease or changes in functioning. Healthy aging facilitates the continuation of wellbeing as health and abilities change through enabling people to actively shape their lives and continue to live out their values as those capacities transform. Experiencing the human-animal bond, the bi-directional and dynamic relationship between a person and pet, can be a value that shapes aging individuals’ choices, preferences, and environment.

### Healthy aging and pets

In the United States, just over half of people ages 50 and older live with at least one pet (Mueller et al., 2018). Pet ownership in older adulthood has been associated with better physical and mental health as well as social connectedness. Reviews of the empirical literature concluded some of the strongest evidence for the association between pet ownership and health are the positive impact of pets on cardiovascular health and physical activity (Gee and Mueller, 2019; Obradović et al., 2020). Pet ownership has also been associated with better executive and cognitive functioning (Branson et al., 2016; Friedmann et al., 2020). While the findings on the relationship between pet ownership and depression are mixed (Gee and Mueller, 2019), pet ownership can have a positive impact on mental health outcomes for those who are socially isolated or have experienced personal loss (Stanley et al., 2014; Carr et al., 2020). Pets can also facilitate interpersonal interactions and foster a sense of community (Wood et al., 2017; Carver et al., 2018). However, pet ownership (i.e., having a pet in the home) provides a limited understanding of the impact of pets on health and wellbeing. The human-animal bond, the dynamic and bidirectional relationship with an animal companion, can be a significant aspect of daily life.

Older adults consistently report their pet is a meaningful and valued relationship (Obradović et al., 2020). Enjoyment and companionship remain the most common reason for owning a pet throughout adulthood (Friedmann et al., 2020). Though the strength of the bond with a pet does not differ between adults of different ages, how the bond impacts daily life may transform as life circumstances, resources, and abilities change (Bibbo et al., 2019). The relationship with a pet is unique as pets are necessarily dependent upon their person for their basic needs. This opportunity to care for another can be an influential source of structure and motivation. Caring for a pet can provide routine and purpose to daily life (Gan et al., 2019). Further, older adults have repeatedly cited their pet’s dependance on them as a factor in health behaviors and routines. The need to care for and be present with a pet has been found to be a motivator for recovery from acute conditions such as stroke, as well as the management of chronic physical and mental health conditions (Peel et al., 2010; Johansson et al., 2014; Janevic et al., 2019; Young et al., 2020).

The provision of care for a pet can continue to contribute to quality of life for pet owners living with dementia (Opdebeeck et al., 2020). People living with Alzheimer’s disease describe pets as providing an opportunity to connect with another, as the bond does not require spoken language and the interactions are non-judgmental (Shell, 2015). While the routines and interactions with a pet are likely to change as the dementia progresses, pets can remain a valued companion (Connell et al., 2007). However, another person must be able to provide for the wellbeing and welfare of the pet as functional abilities change.

Care partners (i.e., family or friend caregivers) are essential to the health and wellbeing of millions of older adults living with and without dementia (AARP and National Alliance for Caregiving, 2020). When the person requiring care is a pet owner, care partners are likely to take on pet care responsibilities (Bibbo and Proulx, 2018). These responsibilities can include basic pet care tasks (e.g., feeding and providing water), as well as taking the pet to the vet and ensuring pet care supplies are in the home. The number of pet care tasks and responsibilities taken on by a care partner are higher when the person requiring care has a greater level of impairment. Pets can substantially impact the time, energy, and resources a care partner devotes to this critical role.

The promotion of healthy aging also relies on healthcare and social service professionals. Though there is not a great deal of existing literature on pet issues encountered by these professionals, there is evidence that addressing the topic of pet ownership can promote honest and productive communication with patients and clients (Hodgson et al., 2017, 2020). These discussions can also uncover risks and benefits to patients’ health. Clients’ pets may also directly impact practitioners who enter their clients’ homes. These pets may be a stressor and may even present a safety risk (e.g., dog bite) (Muramatsu et al., 2019; Owczarczak-Garstecka et al., 2019). A recent meta-synthesis of home health aides’ descriptions of their own occupational health identified pets as an environmental stressor (Grasmo et al., 2021). Five of the 27 studies included in the analysis described both the impact of the pet on the physical environment as well as pets’ behavior as stressful aspects of the job. Therefore, addressing clients’ pet ownership and the bond with those pets may have benefits for both the clients and the professionals themselves.

### The current study

This work was the first objective of a larger investigation to understand the benefits, challenges, and resources of pet ownership in order to promote healthy aging in healthcare and community environments. The study was based on the stress process model that conceptualizes stress as stemming from interrelated factors, including people’s individual characteristics, encountered stressors, and support resources, each of which impact health and wellbeing outcomes (Pearlin et al., 1990). The first objective of this work was to identify the issues stemming from pet ownership encountered by healthcare and social service organization professionals working directly with older adults, people living with dementia, and their care partners. This initial step was crucial as the focus of the extant literature on the impact of pet ownership has concentrated on the resources pet ownership can provide in older adulthood and few have provided the simultaneous provision of resources and stressors (Obradović et al., 2020). Uncovering the challenges of pet ownership will provide a more complete understanding of pet ownership in older adulthood and allow for the construction of theoretically based models on the impact of pet ownership in older adulthood.

Focusing on the experiences of professionals acknowledges professionals’ essential role in healthy aging and may provide a more objective understanding of pet ownership challenges. The emotional bond with a pet may obfuscate negative aspects of sharing the home with an animal companion. Though professionals may be pet owners, they likely do not share a close reciprocated bond with clients’ pets. Further, pets can directly impact professionals (Grasmo et al., 2021) and the relationship with clients (Hodgson et al., 2017, 2020). Identifying the challenges of pet ownership will not only support healthy aging, doing so may improve practitioners’ effectiveness and their own wellbeing.

The first research question in the current study asked whether professionals had encountered issues related to pet ownership in their work with older adults, people living with dementia, and/or care partners. Further, it identified each issue and its source, whether the issue was mentioned to the professional or if the professionals themselves addressed the issue with clients. A hypothesis predicted that professionals who entered homes would be more likely to encounter issues than professionals who did not enter the home as part of their work with clients. The next question asked about the routine experiences of pet ownership. Specifically, what proportion of clients had been pet owners, and did the professionals ask their clients about pet ownership? A final research question asked whether the professionals’ held different attitudes toward pet ownership for different groups (i.e., people general, older adults, and people living with dementia).

## Materials and methods

The study procedures were approved by the Benjamin Rose Institute on Aging’s Institutional Review Board. Institutional Animal Care and Use Committee approval was not sought as the study procedures did not involve non-human participants.

### Participants

The Benjamin Rose Institute on Aging is a project partner in a Geriatric Workforce Enhancement Program (GWEP) funded in 2019 by the Health Resources and Services Administration and led by Northeast Ohio Medical University. The aim of this and other GWEP programs is to improve health outcomes for older adults through educating medical professionals within and outside of geriatrics (American Geriatrics Society, 2022). The project partners include primary care providers, academic institutions, and community-based organizations. The current project capitalized on this network to create an interdisciplinary sample of professionals working with older adults, including those living with dementia, and care partners.

Recruitment activities were influenced by the COVID-19 pandemic. The project was introduced to the GWEP project partners through online meetings and email. Benjamin Rose staff directly emailed contacts at each GWEP partner agency to introduce the project and ask for internal dissemination of an email invitation including a link to the survey and a PDF of the project flier/postcard (originally to be printed and distributed in workplace mailboxes). In addition, study staff reached out to other organizations in the Cleveland area serving older adults (e.g., Cuyahoga County Division Adult Protective Services). Participants were invited to enter a drawing for one of four $50 VISA gift cards upon completion of the survey.

Recruitment activities began in the late winter of 2021 which coincided with the distribution of the COVID-19 vaccines to vulnerable populations. Out of respect for the geriatric workforce, we delayed further activities until the late spring. The survey was closed at the end of July 2021.

A total of 598 surveys were completed, and 136 of those were excluded from analysis. Twenty-five surveys only contained demographic data due to an error in survey design and 111 of respondents did not directly work with the target populations (individuals who had retired and had worked with those populations in the past were included *n* = 7). The analyses included data from 462 surveys.

### Instrument

A one-time anonymous online survey hosted by SurveyMonkey was used to collect all data. Paper surveys were available, but none were requested. The survey contained five sections: (1) Demographics, (2) Professional experiences, (3) Pet ownership, (4) Pet ownership issues, and (5) Open-ended items on the benefits, drawbacks, and resources of pet ownership. The current study focused on the results from sections one through four of the survey.

Demographic items included age, gender identity, race/ethnicity, and education. The employment section asked about current and past professional experiences working with the target populations (e.g., current job title, type of agency, length of time in current position and profession). The job title item was an open-ended response. The agency item asked respondents to select from one of the following: Adult Protective Services, Health Care (e.g., primary care, specialty care, hospice, and palliative care), Housing, Home Health or Caregiving Services, Long-term Care (e.g., nursing home and assisted living facility), Mental Health Services, Social Services (e.g., social work and community service organizations), or Other. Participants who selected “other” were asked to write in the type of agency. One item asked participants to select all the populations they worked directly with (i.e., older adults, people living with dementia, and caregivers/care partners) and another asked whether they went into people’s homes as part of their job.

The professional experiences section also included items specific to pet-owning clients. The instructions for these items asked participants to think of all their experiences working with these populations, not just those experienced in their current position. The first item asked whether they asked the people they work with about pet ownership. The next item asked participants to approximate the percentage of clients who had been pet owners, and the next asked what species those pets (if any) had been. One 5-point Likert-style item asked, “In general how do you feel about older adults being pet owners?” and another asked the same about people living with dementia being pet owners, with response choices ranged from 1, = *Extremely Unfavorable* to 5 = *Extremely Favorable*.

The pet ownership section began with a similar item asking “What is your overall attitude toward pet ownership” using the same 5-point scale. Items also asked about current and past pet ownership, including species and number of pets in their home. These items were asked after the employment section to lessen the potential influence of personal experiences with pets on the recalling of professional experiences.

#### Pet ownership issues

The pet ownership issues section included a matrix with rows consisting of 12 issues stemming from pet ownership. The matrix was developed through reviewing the literature and eliciting feedback from geriatric professionals. First, an initial list of issues was developed by condensing a list of 25 pet care tasks and activities used in a previous investigation of pet care performed by family and friend caregivers (Bibbo and Proulx, 2018). For example, the separate tasks of “give prescription medicine,” “give preventative medicine,” “schedule veterinary appointments,” “take to veterinarian,” were condensed as “routine medical care.” The initial issues were limited to the immediate tasks. Specific concerns such as falling and the need for planning (e.g., due to an older adult transitioning from home to long-term care) were added based on pet-related issues in the described in the literature (e.g., Enders-Slegers and Hediger, 2019).

A survey that included 10 issues was presented to two groups of geriatric professionals, trained SHARE counselors and members of a GWEP subcommittee. SHARE for Dementia is an evidence-based care-planning program developed and licensed by the Benjamin Rose Institute on Aging that provides professionals with the tools they need to help families facing an early-stage dementia diagnosis (Orsulic-Jeras et al., 2019). Sixteen SHARE counselors attended an informational session on pet care and care planning in which they also discussed issues they had encountered. Feedback was provided by members of the GWEP subcommittee via email. Two issues were added based on the feedback: getting pet care items into the home and planning for the pet due to the death of the older adult.

The final survey asked participants to indicate if they had ever encountered the following issues: Basic care (e.g., feeding, grooming, and managing waste); Exercising the pet (e.g., dog walking); Routine medical care (e.g., regular vet check-ups and giving medication); Getting pet care items (e.g., food, cat litter, and other supplies into the home); The financial aspect of pet ownership (i.e., the costs associated with pet ownership); Concerns about the pet’s health (e.g., current or future); Concerns about the pet’s behavior (e.g., aggression and house soiling); Concerns about falling due to the pet or pet items; Rehoming or relinquishing the pet; Planning for the pet due to an emergency (e.g., hospital admission, disaster preparation); Planning for the pet due to the older adult having to move (e.g., transitioning from the home to long-term care); and Planning for the pet due to the death of the older adult.

The columns of this matrix listed whether and who had raised each issue. Participants were asked to select all that applied: “I have not encountered the issue,” “Older adult raised issue,” “Person living with dementia raised issue,” “Care partner/Caregiver raised issue,” “I raised the issue,” “I recognized the issue.” Participants were instructed to select the recognize response option when they had encountered an issue and the issue had not been discussed. A final item asked whether they had encountered any issues not included in the matrix and were asked to describe the issue(s) and who had raised it/them in an open-ended comment box.

### Data analysis

Descriptive statistics were used to analyze all quantitative demographic, employment, and pet ownership data. Job title data were classified into categories created based on the data provided. The first and second authors read the data separately and then worked together to create categories. Data that did not fit into a category was labeled as “other.” When “other” was selected for type of agency, the data were read to determine whether they did fit into the seven *a priori* categories. The first, second, and fifth authors worked together to make these determinations. “Other” was selected when two or more agencies were listed.

#### Pet ownership issues

Binary variables were constructed for each of the 12 issues to measure whether a specific issue had been encountered. When a participant indicated they “had not encountered” the issue the variable was coded with a 0, when one or more of the other response options was indicated the variable was coded with a 1. If no responses had been selected the data was missing for the binary variable. These binary variables were used to determine the proportion of the sample that had an encountered each of the 12 issues. They also allowed for analysis of the number of issues participants had encountered in their work.

Data from the matrix (i.e., an older adult raised the issue, a person living with dementia raised the issue, a care partner/caregiver raised the issue, I raised/recognized the issue) were used determine the proportion of a specific population that had raised the issue to the professional. Only participants who had indicated they worked with the specific population were included in those analyses. For example, only participants who indicated they worked with people living with dementia were included in determining the proportion of people living with dementia who had raised each issue. The binary issue variables were also used to determine the proportion of professionals who had raised an issue.

Independent-samples *t*-tests were conducted to determine differences in the number of issues encountered between those who entered or did not enter clients’ homes. The first series of these *t*-tests determined whether there were differences in the total number of issues encountered, as well as the total number of issues raised by each stakeholder. The Bonferroni correction was used to control for the increased risk of Type 1 error (level of significance set at *p* ≤ 0.0083).

The open-ended responses were initially coded to determine whether each response described an existing code, described a personal rather than a professional experience, or contained a “true other” issue. The first and second authors did this separately and then worked together to reach consensus. Responses could include more than one of the three codes. Only data coded as “true other” was included in the qualitative analysis to determine other issues encountered by the sample.

The first and second authors used a conventional content analysis approach to analyze the qualitative data (Hsieh and Shannon, 2005). No *a priori* codes (i.e., issues) were created, instead each author read through the responses separately multiple times and created codes that emerged from the data. They then met to discuss those codes and reach consensus on the final codes developed from the data. Inter-rater reliability was not determined for this limited data. Instead, the first and second authors independently coded the data and then worked together reach consensus when necessary.

#### Attitudes about pet ownership

Attitudes about pet ownership were compared using a within subjects repeated measures ANOVA. A separate repeated measures ANOVA using the grouping variable of working in clients’ homes was conducted to determine whether going into clients’ homes was associated with attitudes about pet ownership. The Greenhouse–Geisser correction was used as the assumption of sphericity was violated in both analyses.

## Results

### Demographics and pet ownership

The sample was largely female and well-educated (see Table 1). Over eighty percent of the sample identified as White and about two percent identified as a race or ethnicity not included in Table 1 (two identified American Indian/Alaskan Native, Middle Eastern/North African, or “other”; one individual identified as Asian or Asian American and one identified as Native Hawaiian or other Pacific Islander).

**TABLE 1:** Sample demographics.

Characteristic	*n*(%) or *M*(*SD*)
Age (*n* = 452)	53.02 (12.18)
Gender (*n* = 460)	
Female	410(89.13%)
Male	48(10.43%)
Other	2(0.43%)
Race or ethnicity (*n* = 460)	
Black or African American	65(14.13%)
Hispanic or Latino	8(1.74%)
White	384(83.48%)
Another or “Other”	8(1.74%)
Education (*n* = 459)	
High school or GED	31(6.75%)
Some college	39(8.50%
Associate degree	45(9.80%)
Bachelor’s degree	186(40.52%)
Master’s degree	137(29.85%)
Doctoral, MD, or JD degree	21(4.58%)

Almost three-quarters of the sample currently lived with a pet (*n* = 341, 74.78%; data was missing for six participants) with an average of 2.53 pets in the home (*n* = 330, *SD* = 3.82). Dogs were the most common type of pet (*n* = 253, 77.12%), followed by cats (*n* = 158, 46.33%), fishes (*n* = 26, 7.62%), reptiles (*n* = 12, 3.52%), birds (*n* = 9, 2.64%), small mammals (*n* = 7, 2.05%), and horses (*n* = 3, 0.88%). Fifteen (4.40%) reported living with an “other” type of pet. Most people who did not currently live with a pet (*n* = 119) had in the past (*n* = 101, 84.87%).

### Professional experiences

Four hundred and sixty participants (99.57%) provided their current job title. Sixteen codes were developed from these data, along with an “other” category (*n* = 23, e.g., Art Therapist, Editor, Inn Keeper, and Specialist). Ninety participants selected “other” for the type of agency item, and all 90 wrote in the type of agency they worked for. Sixteen of these respondents had selected an existing category and this response (not the qualitative response) was used in analyses. The agency type was changed for one participant who had selected “Social Services” and also written in “APS.” A total of 18 respondents were re-coded as working in a social service agency, including 11 who reported working for an Area Agency on Aging and four who worked for a senior center. Seventeen were recoded into the health care category (e.g., “Dialysis,” “hospice,” and “hospital”). One respondent who described their agency type as “long term care ombudsman” was recoded as long-term care. Table 2 describes how the job type categories were distributed in the seven *a priori* categories along with those who fell into the “other/none” category.

**TABLE 2:** Current type of job and employment experiences by agency categories.

	Adult protective services (*n* = 55)	Health care (*n* = 194)	Housing (*n* = 7)	Home health or Caregiving services (*n* = 12)	Long-term care (*n* = 14)	Mental health services (*n* = 5)	Social services (*n* = 135)	Other or missing (*n* = 40)
Job Type	*n* (%)	*n* (%)	*n* (%)	*n* (%)	*n* (%)	*n* (%)	*n* (%)	*n* (%)
Administration (*n* = 110)	4 (7.41%)	30 (15.54%)	3 (42.86%)	1 (8.33%)	2 (14.29%)	1 (20.00%)	55 (40.74%)	14 (35.00%)
Care/Case Management (*n* = 44)	16 (29.63%)	1 (0.52%)	–	1 (8.33%)	1 (7.14%)	–	20 (14.81%)	5 (12.50%)
Educator (*n* = 5)	–	3 (1.56%)	–	–	–	–	–	2 (5.00%)
Legal (*n* = 4)	–	–	–	–	–	–	–	4 (10.00%)
Maintenance (*n* = 4)	–	3 (1.56%)	–	–	–	–	1 (0.74%)	–
Medical Doctor (*n* = 6)	1 (1.85%)	4 (2.07%)	–	–	–	–	1 (0.74%)	–
Nurse (RN/LPN) (*n* = 76)	1 (1.85%)	53 (27.46%)	–	3 (25.00%)	2 (14.29%)	–	13 (9.63%)	4 (10.00%)
Nursing Assistant (*n* = 26)	–	23 (11.92%)	–	1 (8.33%)	2 (14.29%)	–	–	–
Ombudsman (*n* = 5)	–	–	–	–	3 (21.43%)	–	2 (1.48%)	–
Patient Care (*n* = 4)	1 (1.85%)	–	–	–	–	–	3 (2.22%)	–
Physical Therapist (*n* = 2)	–	1 (0.52%)	–	1 (8.33%)	–	–	–	–
Retired (*n* = 16)	–	9 (4.66%)	–	–	1 (7.14%)	–	1 (1.48%)	5 (12.50%)
Social Work/LISW (*n* = 66)	18 (33.33%)	23 (11.92%)	1 (14.28%)	–	1 (7.14%)	4 (80.00%)	19 (14.07%)	–
Spiritual Care (*n* = 6)	–	5 (2.59%)	–	–	1 (7.14%)	–	–	–
Supervisor (*n* = 27)	10 (18.52%)	4 (2.07%)	–	–	–	–	12 (8.89%)	1 (2.50%)
Volunteer (*n* = 35)	–	29 (15.03%)	–	3 (25.00%)	1 (7.14%)	–	–	2 (5.00%)
Other or Missing (*n* = 26)	4 (7.27%)	6 (3.09%)	3 (42.86%)	2 (16.67%)	–	–	8 (5.93%)	3 (7.50%)
Employment Experiences	*n* (%) or *M* (*SD*)	*n* (%) or *M* (*SD*)	*n* (%) or *M* (*SD*)	*n* (%) or *M* (*SD*)	*n* (%) or *M* (*SD*)	*n* (%) or *M* (*SD*)	*n* (%) or *M* (*SD*)	*n* (%) or *M* (*SD*)
Full-time Position	53 (96.36)	148 (76.29)	6 (85.71)	9 (75.00)	9 (64.29)	4 (80.00)	124 (91.85)	*
Goes into Clients’ Homes	54 (98.18)	110 (56.99)	3 (42.89)	9 (75.00)	7 (50.00)	5 (100.00)	91 (67.91) (*n* = 134)	*
Years in Current Position	9.21 (7.40)	9.79 (7.98) (*n* = 186)	4.05 (4.02)	13.75 (13.75)	7.69 (8.91) (*n* = 13)	13.80 (15.80)	8.97 (8.39) (*n* = 132)	*
Years Working with Population	16.63 (10.34)	16.73 (11.51) (*n* = 189)	6.36 (6.86)	21.58 (12.24	15.36 (12.34)	21.80 (18.34)	16.93 (11.26) (*n* = 133)	*

*Employment experience variables are not presented as they include multiple agency types. These results are available upon request.

Three new agency categories were created: government (*n* = 9, e.g., “city government,” “State Unit on Aging”), education (*n* = 3, i.e., “Education” and “University”), and legal (*n* = 3, e.g., “Law Firm,” and “Law Practice”). The types of jobs represented in the government category were administration (*n* = 8) and legal (*n* = 1). One participant who worked in education described their job as educator and the other two selected nurse. Two people working in a legal agency also described their job as legal, and the other had selected supervisor.

Table 2 also provides the employment experiences by the seven *a priori* agency categories. Participants (*n* = 448) had been in their current positions for an average of 9.61 years (*SD* = 8.83) and working with the target populations (*n* = 454) for an average of 16.93 years (*SD* = 11.49). Three-quarters of the sample worked with more than one of the target populations (worked with two: *n* = 60, 12.99%; worked with all three: *n* = 281, 60.82%). Four hundred and twenty-two (91.34%) worked with older adults, 321 (69.48%) worked with people living with dementia, and 334 (72.29%) worked with care partners. Seven (1.52%) indicated they did not work with any of the populations on the specific item; however, responses elsewhere in the survey indicated they had in the past. The professionals (*n* = 452) were largely working full-time in this position (*n* = 379, 83.84%; part-time *n* = 73, 16.15%) and about 65% (*n* = 298, 64.92%, data missing for three participants) went into the homes of the older adults and/or families they worked with as part of their job.

### Working with pet owners

About 40% of participants indicated they asked the older adults they worked with about pet ownership (*n* = 188, 41.23%, data missing for six participants). One-third (*n* = 152, 33.33%) sometimes asked about pet ownership, 13.16% (*n* = 60) let the older adult bring up the topic, and 12.28% (*n* = 56) did not ask about pet ownership. A *post hoc* descriptive comparison was conducted to determine whether there was a difference in asking about pet ownership between those do did and did not enter the homes of their clients. Table 3 provides the results of this descriptive comparison. A chi-square test was run following the descriptive comparison which indicated there was a significant relationship between entering homes and asking about pet ownership, *X*^2^ (3, *n* = 453) = 30.11, *p* < 0.001.

**TABLE 3:** Asking about pet ownership based on entering clients’ homes.

Do you ask about older adults’ pet ownership?	Enter homes (*n* = 293)	Do not enter homes (*n* = 160)
	*n* (%)	*n* (%)
Yes	144 (49.15%)	43 (26.89%)
Sometimes	95 (32.42%)	56 (35.00%)
I let them bring it up	31 (10.58%)	28 (17.50%)
No	23 (7.85%)	33 (20.63%)

Respondents (*n* = 441) estimated just under half of older adults they worked with had been a pet owner (*M* = 46.29%, *SD* = 21.03%). Dog ownership was the most commonly encountered type of pet ownership encountered (*n* = 429, 92.86%), followed by cat (*n* = 408, 88.31%), bird (*n* = 147, 31.82%), fishes (*n* = 65, 14.07%), small mammal (*n* = 29, 6.28%), reptile (*n* = 22, 4.76%), horse (*n* = 19, 4.11%), and “other” species (*n* = 15, 3.25%). A *post hoc* independent samples t-test indicated there was not a significant difference in the estimated percentage of client pet ownership between those who entered the home (*M* = 45.82, *SD* = 20.82) and those who did not enter clients’ homes (*M* = 47.24, *SD* = 21.63), *t*(436) = −0.67, *p* = 0.506.

### Issues encountered

**FIGURE 1:**
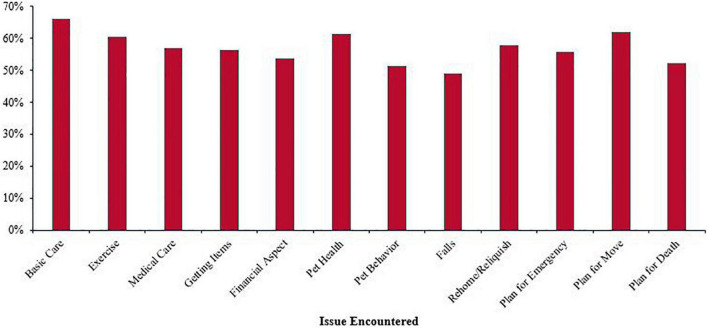
Percentage of professionals who encountered a specific issue (*N* = 462).

Ninety-six (20.78%) of the professionals had not encountered issues related to pet ownership in their work, while a similar percentage reported encountering all 12 issues (*n* = 93, 20.13%) (*M* = 6.81, *SD* = 4.54). Figure 1 presents the percentage of professionals who encountered each of the 12 issues regardless of who had raised the issue. The figure also includes the professionals who recognized the specific issue and may or may not have raised it in their work as these include data from the matrix (i.e., ‘I raised the issue,’ and ‘I recognized the issue’) and qualitative responses (responses were not duplicated). The three issues that were encountered by the most professionals were basic care (*n* = 305, 66.02%), followed by planning for the pet due to moving (*n* = 286, 61.90%), and concerns for pet health (*n* = 283, 61.26%).

**FIGURE 2:**
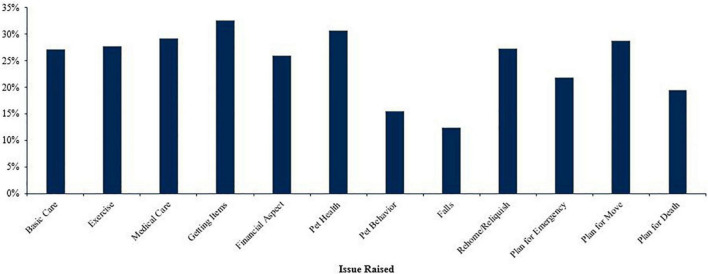
Percentage of professionals who encountered an older adult raising each issue (*n* = 422).

The professionals who worked with older adults (*n* = 422) reported that the older adults themselves had raised an average of 2.98 issues (*SD* = 3.54). Figure 2 illustrates the percentage of professionals who reported working with older adults who had an older adult raise a specific issue. The issues the professionals had older adults raise most often were getting pet care items (*n* = 137, 32.46%), concerns about pet health (*n* = 129, 30.57%), routine medical care for the pet (*n* = 123, 29.15%), and planning for the pet due to moving (*n* = 121, 28.67%). The issues raised least often by the older adults were concerns about pet behavior (*n* = 65, 15.40%), and concerns about falling (*n* = 52, 12.32%).

**FIGURE 3:**
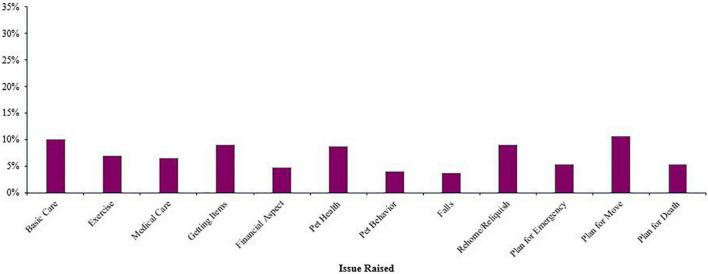
Percentage of professionals who encountered a person living with dementia raising each issue (*n* = 321).

The professionals who worked with people living with dementia (*n* = 321) reported that the individuals raised very few of the 12 issues (*M* = 0.84, *SD* = 2.04) (see Figure 3). The professionals had most often encountered persons with dementia raising the issues of planning for the pet due to moving (*n* = 34, 10.59%), followed by basic pet care (*n* = 32, 9.97%), getting pet care items into the home and rehoming or relinquishing the pet (both *n*s = 29, 9.03%). Concerns about pet behavior (*n* = 13, 4.05%) and about falling (*n* = 12, 3.73%) were the least often raised.

**FIGURE 4:**
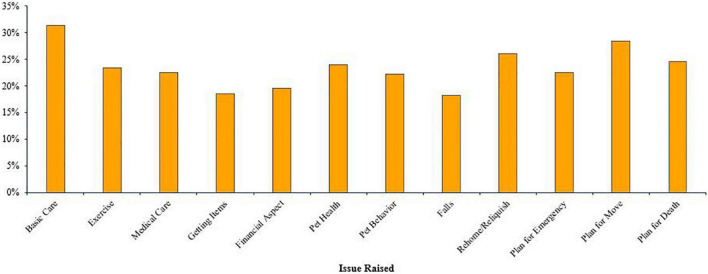
Percentage of professionals who encountered a caregiver raising each issue (*n* = 334).

The professionals who worked with care partners/caregivers (*n* = 334) reported that the care partners raised an average of 2.81 of the 12 issues (*SD* = 3.74) (see Figure 4). Basic pet care was the most commonly raised of the issues (*n* = 105, 31.44%), followed by planning for the pet due to moving (*n* = 95, 28.44%), and rehoming or relinquishing the pet (*n* = 87, 26.05%) was the third most often raised. The three least often raised by care partners were the financial aspect of pet ownership (*n* = 65, 19.56%), getting pet care items into the home (*n* = 62, 18.56%), and concerns about falling (*n* = 61, 18.26%).

**FIGURE 5:**
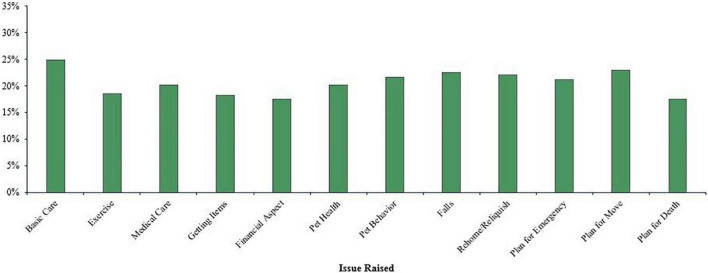
Percentage of the professionals who raised a specific issue.

The professionals themselves reported raising an average of 2.47 of the 12 issues (*SD* = 3.62) in their work with clients (see Figure 5). The professionals most often raised the issues of basic pet care (*n* = 115, 24.89%), followed by planning for the pet due to moving (*n* = 106, 22.94%), and concerns about falling (*n* = 104, 22.51%). Getting pet care items into the home (*n* = 84, 18.18%), the financial aspect, and planning for the pet due to the older adult’s death (*n* = 81, 17.53%) were the issues least often raised by the professionals.

**FIGURE 6:**
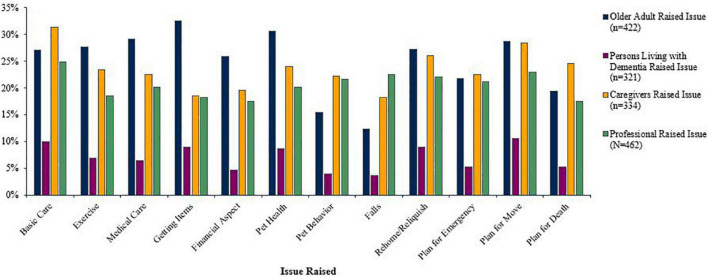
Percentage who raised each issue by group.

Figure 6 illustrates the proportion of each group that raised each issue.

#### Other issues encountered

Ninety-four professionals (22.07%) indicated they had encountered another type of issue not included in the matrix, while 110 (23.81%) respondents provided an answer to the open-ended item asking for a description of other issues encountered in working with the target populations. Six of these responses did not contain codable data (i.e., “I raised it.” and “Older adult”). Codable data was categorized as describing an existing issue, a “true” other, or a personal experience (i.e., describing an experience with family or friends rather than a professional experience). Twenty-two of the 104 (21.15%) responses included both existing and “true” other issues, and one (0.96%) included all three categories. Fifty-nine (56.73%) contained data describing one of the 12 existing issues [e.g., “Most elders are concerned about what will happen to their pet(s) when they pass.” was coded as older adults raising the issue of planning for the pet due to death], and three (2.88%) contained personal experiences which were not included in analyses (e.g., “My mother has primary progressive aphasia, and getting to see our dogs in person and even just pictures helps her communicate.”). Sixty-nine (66.34%) included “true” other issues. Eleven new issues came from these data. A “miscellaneous” code was used to denote data that did not refer to specific issues relating to pet ownership (e.g., “I am glad to see more businesses allowing pets in their stores. I have seen many older people take their animals into stores with them.”). The 11 new codes were: Unsanitary conditions, Policies within facilities, Refusing care, Separation, Unaware of needs, Hoarding, Impeding Care, Abuse, Dead pet, Housing, and Caregiver burden. Information on whether these issues had been raised, or who had raised these issues, was not always provided in the response.

Fourteen professionals described pets creating unsanitary home environments, primarily due to an accumulation of pet waste. Foul odor was a common consequence of unsanitary conditions and often brought up by the professionals or care partners (e.g., “[the older adults] almost never talk about it….they are either used to the smell or are too proud or embarassed [*sic*] and/or don’t want to have to give up their pets.”). Having too many pets in the home also led to unsanitary conditions, and these conditions could also impede access to needed care (e.g., “… home health aides do not want to work in an environment with extreme pet hair, pet waste or smell.”). One respondent described encountering flea infestations “many times.” These unsanitary conditions created “an unhealthy environment for individual/family/animals” who lived in, as well as the professionals who worked in, the home.

Policies within facilities, refusal of care, and separation did overlap with the *a priori* issues of planning for the pet due to an emergency and planning for the pet due to the older adult having to move. However, these new codes each addressed a factor shaping (i.e., policies), or resulting from (i.e., refusal of care) these issues, or a combination of the two (i.e., separation). Seven participants described policies in long-term care facilities being an issue. Most of these respondents identified themselves as working within these facilities and having seen issues arising from residents who had previously been pet owners not being able to continue living with pets due to policies or costs (e.g., “I work with assisted living and there is sometimes an up front cost and sometimes a monthly fee tacked onto the rent. Residents only have $50.00 per month for their personal needs.”) and not having pet visitation policies (e.g., “Facilities that refuse to allow pets to be brought in to visit previous owners or residents who love and miss their former pets.”). Some of these comments also included the need for policies to ensure safety when pets did visit (e.g., “Helping families know that pet visits are great but pets must be vaccinated and on a leash.”) and alternatives to pet ownership such as a “community pet.”

Refusing care, described by 12 participants, was not only the refusal of transitioning out of the home, but also refusing needed acute medical care and had been brought up by older adults, their care partners, and the professionals. The refusal of needed care could have negative implications for the health of both the person and their pet (e.g., “Client refused to seek medical attention because she was afraid to leave her pet alone. I tried to offer making arrangements for the dog to be taken care of but the client refused. The client was unable to care for herself or the dog and the dog’s health was failing as well.”). Refusal of care also included refusing to leave unsafe living environments due to fear of not being able to remain with the pet (e.g., “In my experience, a lot of seniors will remain in a poor home environment/poor home care plans, in order to assure that the pets in the home are not removed and or displaced.”). One professional described the impact on unhoused pet owners, “There are barriers for homeless older adults seeking shelter as most shelters do not allow pets. This leaves the individual with a difficult choice and they often will refuse shelter rather than give up the pet.”.

Though separation was only explicitly mentioned in eight of the comments, the issue – people not wanting, resisting, or refusing to separate from their pet – underlies many of the issues described above. Professionals themselves recognized the potential negative impact of separation on the owners (e.g., “To separate the pet from the owner would be a huge loss”), including those living with dementia, “Those with dementia that cannot maintain their pets due to their memory loss but they still do not want to have their pet taken or move and leave them behind even when they cannot take care of them (even though they may not realize they cannot).”

The last excerpt also illustrates the issue of owners being unaware of needs, such as basic care and medical pet care. Older adults or people living with dementia being unaware of their pets’ needs was mentioned by ten participants. These situations were exclusively recognized by the professionals or care partners and were distinct from the physical or financial inability to provide needed care. The impact on pets ranged from “not being up to date with vet appointments” to unintentional neglect and/or abuse of the pet (e.g., “I’ve experienced a person living with dementia forgetting they had a pet where it became a neglect situation.”). Responses also linked this issue to an older adult neglecting their own needs (e.g., “Dogs often neglected and the owner does not seem to see it… as they themselves are not taking care of their own human needs.”). One professional described a situation where a person living with dementia forgot they had a pet in the home and another described a situation where a person living with dementia forgot their family had removed the pet from the home (“Three weeks after family member took dog away, person living with dementia said, ‘I have a dog here somewhere, but I don’t know where he is right now’.”) These situations were often due to cognitive limitations, but also due to changes in sensory functioning (“I have encountered older adults who is unable to see, smell or acknowledge the problems with their pet.”). Unawareness of pet needs had also led to unsanitary home environments.

Hoarding of companion animals was described by eight individuals (e.g., “I have had multiple clients with pet hoarding issues.”). One participant reported that “Housing and/or City Inspectors” had raised the issue. All individuals who had encountered it said that they themselves had recognized the issue.

Pets impeding access to care and/or services were described by five individuals. This included the pet’s behavior creating a safety issue for the professional (e.g., “Aggressive animals that inhibit the member older adults’ ability to receive services in the home due to the animal – I raised the issue. Older adult aware but chooses the animal”) as well as pet ownership causing a negative impact on the home environment (“… home health aides do not want to work in an environment with extreme pet hair, pet waste or smell”). This issue was exclusively raised by professionals.

Four participants described abuse that occurred to either the older adult or the pet. Two respondents described the pet being abused in order to inflict abuse on the older adult (e.g., “Sometimes in abusive situations, the individual that is exploiting the older adult will use the pet as a means of controlling the older adults. They have threatened to take the pet away or harm the pet.”). One participant stated a person living with dementia had abused their own animal. Whether the abuse was intentional or unintentional could not be determined from the comment (“Person living with dementia neglecting and abusing a 17 y/o dog. not walking, kicking, pushing down, dragging on leash, and not cleaning urine/feces in the home”).

Three participants had encountered situations where a pet had died. One comment focused on the emotional impact of the death and the other two described the consequences of the pet’s physical body. In one situation a colleague had disposed of the pet’s body (“Older adult asked a coworker to come over and arrived to find the dog dead. … Coworker buried the pet for the older adult.”).

Housing issues and caregiver burden were each described by two respondents. One reported housing policies (i.e., “Housing that does not allow pets”) being an issue that had been raised by older adults and their family. The other explicitly mentioned housing issues having an impact on maintaining pet ownership being raised by older adults, themselves, or other professionals (“eviction/foreclosure and lack of stable housing to maintain a pet has occurred.”). Caregiver burden was identified by two participants. They described the extra work or strain that a pet caused a care partner (e.g., “Caregiver fatigue”).

#### Professionals working in homes

Professionals who entered the home as part of their work had encountered more issues than those who did not enter the home (see Table 4). There were no significant differences in the number of issues raised by older adults, people living with dementia or care partners to the professionals (all *p*s ≥ 0.196). However, the professionals themselves raised more issues when they entered the home.

**TABLE 4:** Results of analysis comparing number of issues raised by entering homes.

Stakeholder group	Enter homes		Do not enter homes		*df*	*t*	*p*	Cohen’s *d*
	*n*	*M* (*SD*)	*n*	*M* (*SD*)				
Total issues encountered	298	7.34 (4.51)	161	5.81 (4.44)	457	3.497	0.00052	0.342
Raised by older adults	290	3.13 (3.63)	129	2.69 (3.35)	417	1.185	0.237	0.125
Raised by people living with dementia	235	0.92 (2.18)	83	0.63 (1.64)	190.70	1.297	0.196	0.145
Raised by care partners/caregivers	235	2.92 (3.74)	97	2.48 (3.62)	330	0.972	0.332	0.117
Raised by professionals	298	3.10 (3.91)	161	1.35 (2.71)	429.40	5.593	<0.00001	0.492

### Attitudes about pet ownership

**FIGURE 7:**
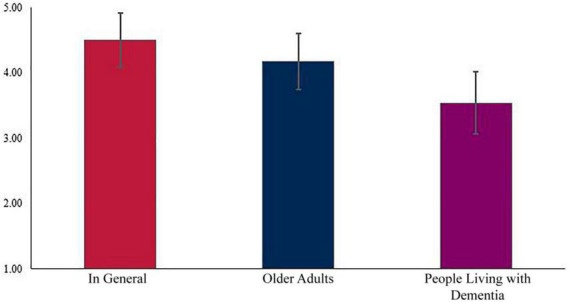
Differences in favorability of pet ownership.

The results of the within-subjects ANOVA indicated favorability toward pet, ownership differed between who owned the pet, *F*(2,807.218) = 254.642, *p* < 0.001 (see Figure 7). *Post hoc* pairwise comparisons indicated significant differences in favorability about pet ownership. Favorability about pet ownership in general (*M* = 4.50, *SD* = 0.04) was significantly higher than for older adults being pet owners (*M* = 4.17, *SD* = 0.04) (0.32 [95% CI, 0.26–0.40), *p* < 0.001]. Similarly, favorability of pet ownership for older adults was significantly higher than favorability about pet ownership for people living with dementia (*M* = 3.54, *SD* = 0.04) [0.63 (95% CI, 0.55–0.71), *p* < 0.001].

The results of the repeated measures ANOVA found there was no difference in the favorability of specific groups being pet owners based on whether a professional entered the home or not as part of their work, *F*(1,1.777) = 0.771, *p* = 0.400.

## Discussion

The majority of professionals in this sample had encountered issues related to pet ownership in their work with older adults, people living with dementia, and care partners. There were differences in who raised each issue, which may indicate the salience of issues varies between individuals. The professionals and care partners raised the issues of pet behavior and the risk of falls more commonly than the pet owners. Other issues, specifically, basic pet care, planning for the pet due to a move or emergency, and relinquishing or rehoming a pet were raised by all types of clients and the professionals themselves.

Older adults raised more issues to the professionals than any other group. Their concerns focused on the welfare of their pet as they were the group to most often raise the issues of getting pet care items into the home, concerns about the pet’s health, routine medical care, and exercise. Older adults also broached the financial aspect of being a pet owner more often. This issue has a direct impact on the capacity of an individual to provide for the pet’s physical needs and can underlie the other issues most often mentioned by the older adults.

People living with dementia raised issues much less often than older adults without dementia or any other group of people. People living with advanced dementia may not be aware of issues. Indeed, older adults not being aware of their pets’ needs was an issue that emerged from the open-ended item. Though a lower proportion of professionals had a person living with dementia raise pet-related issues, people living with dementia were similar to other older adults as the issues they most often raised related to their pets’ health and welfare.

The professionals reported that care partners were the group who raised the issue of having to plan for the pet due to the death of the older adult most often. Care partners were also the group that most commonly raised the issue of basic pet care. This may have been due to taking on these responsibilities and the potential impacts those responsibilities can have on the emotional experience of caregiving (Bibbo and Proulx, 2018, 2019). Issues related to planning were also raised by care partners as they are likely linked to having an active role in the older adults’ healthcare and other decision-making.

The professionals were the only group who had concerns about falling within their three most often raised issues. People in the geriatric workforce are likely to be aware of the significant potential consequences of falling for older adults. Falls are the leading cause of death from an accidental injury for people ages 65 and older (Cheng et al., 2016; Burns, 2018). Pets can be a fall hazard for people of all ages, though the likelihood of experiencing a serious injury from the fall is highest in older adults (Stevens and Haileyesus, 2009). Though pets can present an environmental fall hazard, they are most likely to pose a risk for those who are living with the major risk factors for experiencing a fall (e.g., impaired balance and gait, polypharmacy, and previously falling) (Ambrose et al., 2013).

### Other issues professionals encountered

The issues uncovered in the open-ended item provided insights into the interactions between an owner’s functional ability, the home environment, and the health and wellbeing of people and pets. This was exemplified by the issue most often described, pet ownership creating unsanitary conditions in the home. These conditions often arose from changes in the older adults’ physical or cognitive abilities to manage animal waste and led to conditions that were unpleasant, detrimental, and even dangerous for everyone who lived in or entered the home. These environments created a barrier for professionals from entering the home and may have impeded access to needed care. Indeed, pets impeding access to care was described as stemming from unsanitary conditions as well as the pets’ behavior. Further, the results of this study provided evidence that professionals entering homes had to not only encounter, but actively deal with the bodies of dead pets. This is not the first study to find evidence that pets can present a safety for professionals entering a client’s home (Owczarczak-Garstecka et al., 2019), but these findings highlight how the home environment, including the residing pet, can be an obstacle to an older adult receiving needed care.

Pets could also be a reason older adults refused care. This issue was raised to the professionals by older adults, care partners, as well as by the professionals themselves. The refusal of care was directly linked to being separated from the pet. The provided responses focused on the emotional consequences of separation underlying refusal of care, others have found adults may delay or refuse care due to concerns over pet care (Polick et al., 2021). Indeed, planning for the pet due to an emergency such as a hospital stay was encountered by this sample of professionals.

There is ample evidence that pets can be a barrier to housing, particularly for people with limited income (e.g., Huss, 2005; Power, 2017; Toohey and Rock, 2019). This issue was overlooked by this survey but was addressed by two professionals. Policies in long-term and other residential care were described more often, the proportion of the sample working in these agencies may have accounted for this difference. Housing and facility policies are likely to force the issue and/or situation of rehoming or relinquishing a pet (McNicholas et al., 2005; Toohey et al., 2017).

Hoarding or having too many pets in the home was exclusively raised by professionals. “Too many pets” is not synonymous with hoarding, as animal hoarding is a specific manifestation of a diagnosable disorder (Reinisch, 2008). Regardless, both can lead to the same unsanitary conditions described above, though people with hoarding disorder are often unaware of these negative environmental or health effects. The findings also add to the evidence for a linking the hoarding of animals to self-neglect, which has implications for APS professionals (Nathanson, 2009).

The act or threat of physical abuse on a companion animal to control a person is documented in the literature on domestic and interpersonal violence (Vincent et al., 2020). The findings of this study add to the limited evidence that perpetrators of elder abuse may use their victims pets’ as a tool to manipulate and exert control over their victims (Boat and Knight, 2000). The abuse, neglect, and exploitation of older adults is a recognized public health crisis with critical implications for people’s health and safety (Yan, 2019). Understanding how older adults’ pets are exploited in these predatory and pernicious acts can help protect the welfare and wellbeing of older adults and their pets.

The professionals encountered unintentional neglect of a pet as the result of older adults being unaware of their pets’ needs and/or health status. These issues were raised by the professionals as well as care partners. These situations had direct negative impacts on the pet as well as the home environment underscoring the need for asking about pet ownership. Changes in functioning can have direct negative impacts on the health of the person, pet, and their shared environment. These negative consequences may lead to a cyclical effect with the negative impacts of the older adults’ health leading to an unsafe environment for them and their pet, which in turn leads to more functional decline in the older adult.

Spending more hours actively providing care is a significant risk factor for experiencing caregiver burden (Adelman et al., 2014). Previous work with care partners of pet owners found they took on more pet care tasks and activities when the older adult required more assistance (Bibbo and Proulx, 2018). The professionals had encountered caregiver burden as a result of older adults’ pet ownership. How pet care impacts the emotional experiences of caregiving is likely complicated and may be moderated by the care partner’s relationship with the older adult (Bibbo and Proulx, 2019).

### The impact of entering the home

The hypothesis that professionals who entered clients’ homes would be more likely to encounter issues was supported by our findings. The professionals were no more likely to have an older adult, person with dementia, or a care partner raise these issues, only the professionals raised more issues related to pet ownership when they themselves entered clients’ homes. While there was no difference in the estimated percentage of pet ownership between those who entered and did not enter clients’ homes, those who entered the home were more likely to ask clients about pet ownership. These differences could directly stem from their previous experiences of encountering pets inside the home. Indeed, entering a home environment can provide professionals insights to their clients’ lives that cannot be observed in an office setting. It is important to remember that pet issues exist regardless of whether a professional enters the home.

### Attitudes about pet ownership

The sample was very favorable to pet ownership in general. However, they were significantly less favorable about older adults being pet owners, and even less favorable about pets being owned by people living with dementia. While people who entered homes were more likely to recognize and raise issues stemming from pet ownership in their work with these groups, going into the home was not associated with favorability toward pet ownership. The results of this study cannot provide any insight into the specific reasons for these differences. Possible reasons include the number of years of working with these populations along with the experiences of coworkers and in the professionals’ personal lives. Indeed, some of the professionals shared personal experiences they had with older adults in their families (though these data were not included in analyses).

### Limitations

This was not a representative sample of the geriatric workforce. Our methodology led to a convenience sample which severely limits the generalizability of these results. A probability sample would have allowed for a more accurate understanding of the issues encountered by the geriatric workforce. The results of our convivence sample provides evidence that issues are encountered, but we cannot generalize about what issues are encountered nor their prevalence. The generalizability is also limited due to the high rate of current and past pet ownership in the sample. Prior to the COVID-19 pandemic, approximately 60% of United States households included a pet (Applebaum et al., 2020a). Despite articles in the popular press and findings from the pet products industry (e.g., PetAge, 2021), the rate of pet ownership may not have increased dramatically during the first year of the pandemic (Hoffman et al., 2021). Three quarters of the sample were current pet owners and even more had been a pet owner in the past. The professionals in this sample may have been more aware of issues relating to and stemming from pet ownership due to their own experiences as pet owners.

Though we did not collect data identifying where the participants resided or worked, recruitment activities were concentrated in Northeast Ohio. A national sample would better represent the geriatric workforce. Purposely sampling professionals working in a mix of urban and rural communities of various median incomes and population densities would likely provide different results. Further, Cleveland is one of the most economically disadvantaged cities in the United States (DePietro, 2021). Economic constraints shape people’s environment and choices, and all of which are highly likely to impact the experience of pet ownership. We cannot determine whether these constraints shaped the issues the professionals in our sample encountered. Unfortunately, economic constraints are not unusual in the United States. More research is needed to begin to understand how the complex and significant factors that make up social determinants of health also shape pet ownership and the issues encountered by those who work with aging adults and their care partners.

The clients’ characteristics were not measured, but almost certainly affected the issues the professionals encountered. Individuals’ health status and functional abilities (e.g., mobility, cognitive functioning, and abilities to perform activities of daily living) would all impact the ability to provide care for a pet. Available social and instrumental support are also likely to impact the issues encountered. These factors may also influence the favorability about pet ownership for older adults living with and without dementia.

This study did not examine the experiences of older adults, people living with dementia, or care partners. These experiences are imperative for a comprehensive understanding of the issues encountered and managed by these individuals. Without this information we cannot make any generalizable conclusions about how specific pet ownership issues that may impact healthy aging. However, this aim of this study was to understand the experiences of professionals who worked with these populations. These results can provide a starting point to understanding the issues these three populations deal with, but they do not speak to the experiences of these individuals.

Individuals’ characteristics, including the issues discussed above (e.g., economic resources and health and functioning), are fundamental to the stress process (Pearlin et al., 1990). This study only looked at one element of the stress process model: the stressors. Not only were demographic and situational factors overlooked, but the benefits of pet ownership for older adults living with and without dementia were not addressed. Thus, these results contribute a limited depiction of how clients’ pet ownership is experienced by professionals in the geriatric workforce. These results should not be interpreted in isolation.

Nor do these results provide a comprehensive representation of all issues related to pet ownership that people will encounter when working with older adults and care partners. The *a priori* issues presented to participants largely focused on the pet, while those uncovered in the qualitative data largely addressed issues faced by older adults themselves. A more inductive methodology, such as grounded theory, would likely lead to a more inclusive and complete investigation of these issues.

The survey only asked respondents to estimate the rate of pet ownership and types of pets owned by older adults. We did not ask pet ownership items about people living with dementia or care partners. Though the reason was to lessen the redundancy and burden on participants, we unfortunately lost the opportunity to document evidence of differences of pet ownership rates between these groups.

Not including items about the COVID-19 pandemic may have been another lost opportunity. Despite study procedures being shaped by the pandemic, we made the choice not to include items asking about the topic. The aim of the study was to understand what specific issues stemming from pet ownership professionals have encountered in their work. We hoped to capture experiences that would be not necessarily be common, but probable to come across in working with these populations. Therefore, the items in our survey were asked about all the experiences people had had in the professional lives. There is no doubt that the COVID-19 pandemic has raised important and unique benefits, costs, and concerns for pet owners of all ages, including older adults (Applebaum et al., 2020b, 2021a; Whitehead and Torossian, 2020). Other research has focused on this important topic and provides insight about the impact of this unique historical situation on people, pets, and the human-animal bond.

### Next steps

Encountering and/or raising a concern is only a single aspect of an issue. How issues are addressed and even the willingness of professionals to help with issues related to pet ownership (which may or may not be associated with favorability of pet ownership), are also important. These are likely shaped by the perceived benefits, drawbacks, and resources provided by pet ownership. The second objective of the larger study is to identify specific benefits, challenges, and resources provided by pet ownership and the human-animal bond encountered by professionals working with older adults and their caregivers. These topics were each addressed in the fifth and final section of the survey comprised open-ended items.

The benefits of pet ownership in older adulthood are well-documented (e.g., Gee and Mueller, 2019; Obradović et al., 2020). The bond with a pet can be particularly valuable for older people who are socially isolated or living with physical, cognitive, or psychological challenges (Johansson et al., 2014; Stanley et al., 2014; Shell, 2015; Gan et al., 2019; Janevic et al., 2019; Opdebeeck et al., 2020; Young et al., 2020). These studies are largely based on self-reported outcomes. Understanding the benefits of pet ownership from the perspective of geriatric professionals may provide a more objective depiction these resources. However, the need for objectivity cannot discount or invalidate older adults’ lived experiences. Instead, these two perspectives can complement each other to construct a fuller representation of the instrumental, psychological, and emotional benefits of pet ownership – along with the challenges.

The intention of surveying an interdisciplinary sample was to capture the commonalities between a diversity of experiences in those working in the geriatric workforce. Analyzing data within a specific type of agency can provide information on issues that are specifically relevant to specific professions and/or types of agencies. For example, encountering the deliberate abuse of pets to control older adults is of immediate concern to APS agencies. The need to plan for a pet due to the death of an older adult and the associated issues of rehoming a pet may be important issues for people working in hospice care. The current dataset will provide a beginning to understanding the issues that are unique to specific professions, but more work will be needed to fully understand how pets and the human-animal bond shape the lives of the people served by specific agencies and professions.

Future work should also measure how client characteristics (e.g., physical and mental health, cognitive functioning, living environment, available support) affect issues related to pet ownership. As this was the first study we knew of to investigate this topic we chose to ask what people had encountered at any time in their career. Measuring the demographic, health, and social determinants of health variables of clients will provide a clearer understanding of the issues professionals are likely to encounter in specific circumstances.

The results of this study will be interpreted along with the qualitative results to construct models based on the stress process model identifying the benefits (i.e., outcomes), challenges (i.e., stressors), and resources (i.e., support resources) of pet ownership. Three separate models will be constructed for older adults, people living with dementia, and caregivers. These models will provide a foundation for future research and provide immediate practical applications by creating evidence-based recommendations for geriatric professionals. These recommendations will build upon the evidence that conversations about pets can be used to build rapport with clients (Hodgson et al., 2017, 2020) and motivate older adults in their own health behaviors (e.g., Gan et al., 2019; Janevic et al., 2019). Comprehensive depictions of the impact of pet ownership will enable professionals to successfully navigate the drawbacks and build upon the benefits.

## Conclusion

Many professionals working with older adults, people living with dementia, and their care partners do encounter issues stemming from pet ownership in their work. These issues almost certainly contribute to the negative relationship between the rate of pet ownership and age. The rate of pet ownership decreases dramatically between those aged 50 and 85, while the strength of the bond with a pet does not differ between age these groups (Bibbo et al., 2019). Older adults who live with others are more likely to live with a pet (Friedmann et al., 2020). Having assistance with issues related to pet ownership, including the financial issues, may provide the support necessary for people to continue sharing their lives with pets. The professionals in the varied roles who work with the aging population can, and many do, provide these supports. This support enables aging individuals to continue living with a valued companion and make choices that promote their own healthy aging.

Diversity and inequity must also be addressed in discussions and programs centered on healthy aging (World Health Organization [WHO], 2020). Diversity is not referring to demographics, but the recognition that older adults and people with dementia are individuals with their own unique functional abilities as well as home, social, and community environments. The differential effects of cumulative advantage and disadvantage shape the experiences of older adulthood which directly impact individuals’ functional abilities and environments. These factors also shape the choices and experiences of pet ownership (Applebaum et al., 2021b). The results of this study underscore how the health and wellbeing of people and their pets are linked and shaped by these factors. Including pet ownership in discussions with clients is likely to provide a more complete understanding of the issues that shape their functional abilities, environment, choices, and values.

Asking about pet ownership is a start – doing so acknowledges that pet ownership and/or experiencing the human-animal bond may be a value held by a client. However, policies and programs are necessary to provide the support to enact that value. Policies that allow for the housed and unhoused to maintain their relationship with a pet are needed (Toohey et al., 2017; Ramirez et al., 2022). These policies will need to consider the impact on staff. Including pets into a facility is likely to add work for people in direct-service and facility maintenance positions. Creative solutions, such as creating a position that is focused on pet care issues, may make such policies feasible. Programs are increasingly recognizing that the needs of their clients may also be a need for a client’s pet. For example, home delivered meal programs and food banks are recognizing that food insecurity is experienced by the people and pets within a home (Rauktis et al., 2017). People will forgo food to ensure their pet can eat. Providing pet food makes it more likely that the person will eat. The results of this study provide further evidence that people may prioritize the needs of a pet at the expense of their own.

Programs focused on supporting pet owners by assisting with pet care tasks may provide a solution. Services that provide dog-walking, litter box cleaning, or access to veterinary care can help older adults maintain this important relationship and support healthy aging (Johnson et al., 2002; Cryer et al., 2021). The benefits of these services are likely to extend to care partners and well as the professionals working in the geriatric workforce. The results of this study provide evidence that the issues shaping and stemming from pet ownership in older adulthood are interconnected. Furthermore, these issues not only shape and the health and well-being of aging adults and their pets, but they can also have a direct and significant impact on care partners and professionals in the geriatric workforce. The geriatric workforce is essential for promoting healthy aging. Including issues stemming from pet ownership into procedures, policies, and programs is likely to have positive impacts on those served by and working in the geriatric workforce.

## Data availability statement

The raw data supporting the conclusions of this article will be made available by the authors, without undue reservation, to any qualified researcher.

## Ethics statement

The studies involving human participants were reviewed and approved by Benjamin Rose Institute on Aging IRB. Written informed consent for participation was not required for this study in accordance with the national legislation and the institutional requirements.

## Author contributions

JB led all aspects of the study and manuscript writing. JJ was instrumental in data collection, management, analysis, as well as the interpretation of the results. JD and MS contributed to the development of the study, survey, and conclusions drawn from the results. SN assisted in qualitative analysis and manuscript development. All authors contributed to the article and approved the submitted version.
